# Midline Diastema Closure Using an M Spring in a Young Adult: A Case Report

**DOI:** 10.7759/cureus.30777

**Published:** 2022-10-27

**Authors:** Nikhil Kumar, Pallavi Daigavane

**Affiliations:** 1 Orthodontics and Dentofacial Orthopaedics, Sharad Pawar Dental College, Datta Meghe Institute of Medical Sciences, Wardha, IND

**Keywords:** frictionless mechanics, frenectomy, adjustable loop, malocclusion of teeth, midline diastema

## Abstract

The malocclusion known as maxillary midline diastema frequently occurs. For the management of diastema, a variety of therapy approaches are recommended. This article gives a case report of a female patient, age 24, who underwent treatment for a 4-mm maxillary midline diastema following the extraction of an additional tooth between her upper central incisors. The orthodontic treatment index was minimal (grade 2). The maxillary central incisors were fitted with bonded McLaughlin-Bennett-Trevisi (MBT) 0.018" brackets. A rectangular archwire was used after the circular archwire to ligate and activate the M spring. The diastema was successfully closed after a total of four months of treatment. This approach is effective and efficient and uses minimal inventory. This shortens the length of the orthodontic treatment and conserves valuable chairside time.

## Introduction

A space (or gap) between the maxillary central incisors is alluded to as a midline diastema [1]. Broadbent's description of the medial erupting course of the maxillary lateral incisors and maxillary canines results in proper closure of this area in most children. Regrettably, in some people, the diastema does not close on its own. A diastema between the maxillary central incisors in adults is frequently seen as an esthetic or malocclusion issue [2]. Midline diastema affects approximately 98% of six-year-olds, 49% of 11-year-olds, and 7% of 12- to 18-year-olds. The prevalence rates in adult populations have ranged from 1.6% to 25.4% based on the linear quantification of a diastema, and they have been even higher in younger groups [3]. There is a significant body of evidence suggesting that the manner in which characteristics are passed from generation to generation differs significantly according to racial background. For instance, black people routinely have prevalence rates that are higher than those of white people, Asian people, or Hispanic people [4].

Midline diastema can be caused by various conditions, including proclination of the upper labial segment, a large frenum, a missing tooth, a peg-shaped lateral, midline supernumerary teeth, and self-inflicted disease brought on by tongue piercing [4]. A maxillary midline diastema can develop as a result of the labial frenum's insertion into the alveolar bone's notch, leaving a band of dense fibrous tissue between the central incisors [5]. The border of the bone enclosing each tooth may not extend all the way to the median suture, and the two central incisors may emerge far apart. In such cases, no bone is deposited beneath the frenum. A V-shaped bone fissure develops between the two central incisors, leading to an "abnormal" frenum attachment. It is possible that the midline cleft will never completely close because transseptal fibers do not grow across it [2].

Placek's classification is a morphological-functional classification of the kind of labial frenum attachment with the goal of assisting physicians in identifying functional abnormalities that require care. Mucosal, gingival, papillary, and papillary penetrating frenum attachments are classified according to whether they are placed in the mucogingival junction, the connected gingiva, the interdental papilla, or right up to the palate. In some cases, the frenum is completely absent [5,6]. Treatment consists of finding and getting rid of the underlying cause and then moving the teeth with orthodontics, repairing the damage with an esthetically pleasing composite, managing the prosthetic space with porcelain jacket crowns, and putting in laminates [7].

The purpose of this case study is to show how to manage a midline diastema with basic M-shaped spring and sectional archwire mechanics.

## Case presentation

**Figure 1:**
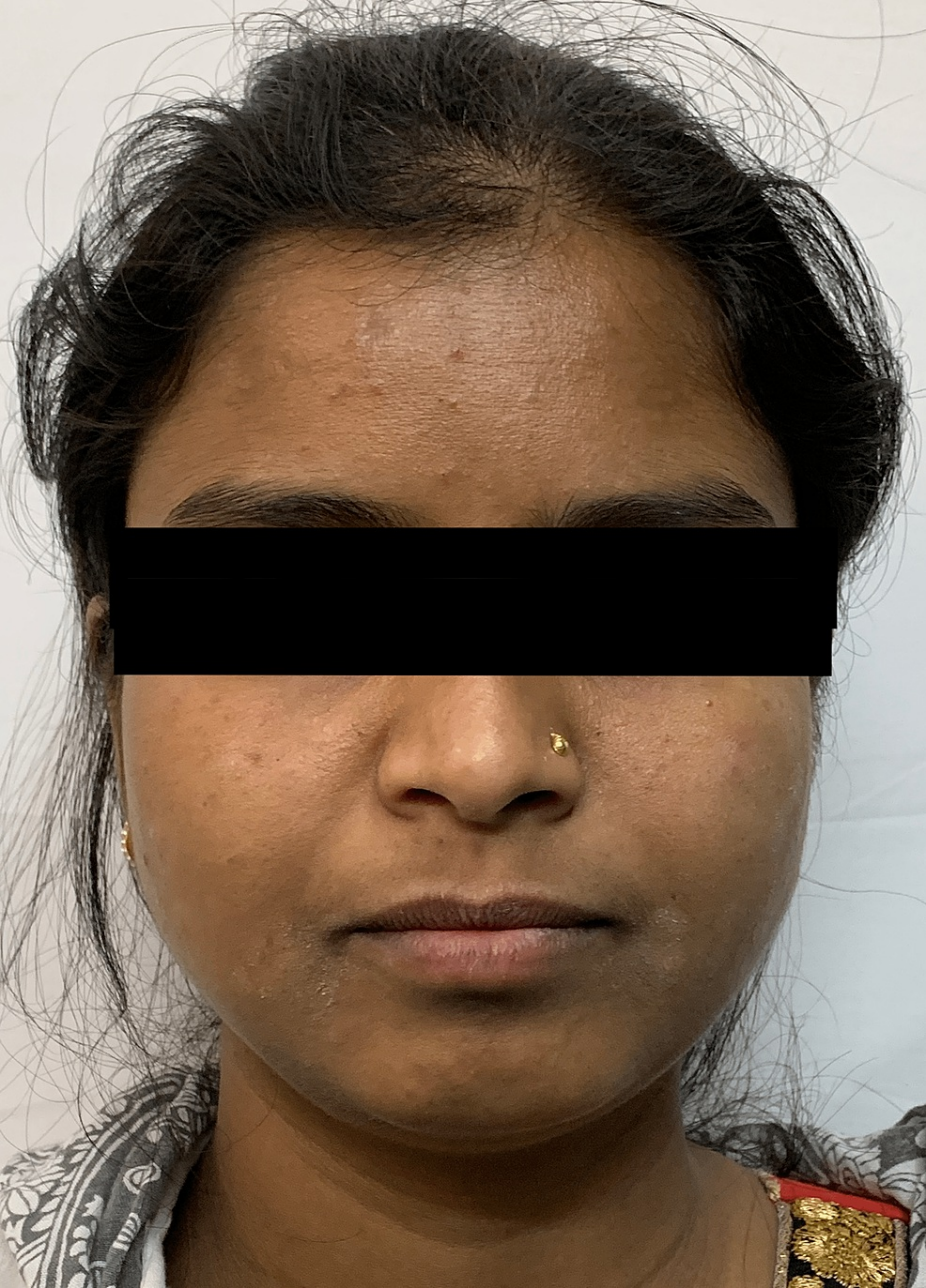
Pretreatment extraoral frontal view

A female patient aged 24 years old presented to the Department of Orthodontics and Dentofacial Orthopaedics. On examination in the orthodontic outpatient department (OPD), the medical history and dental history of the patient were nonsignificant. An extraoral examination revealed that the patient had a straight profile, a mesocephalic head type, a mesoprosopic facial type, competent lips, a straight profile, and a horizontal growth pattern (Figure 1).

**Figure 2:**
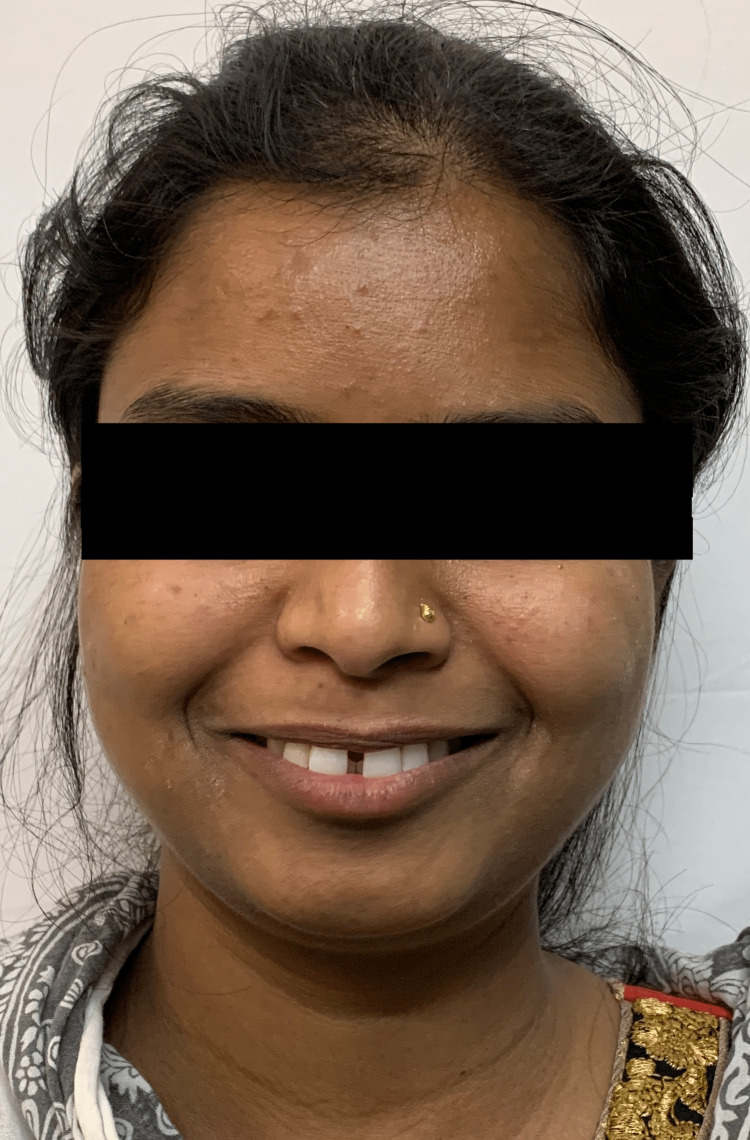
Pretreatment extraoral smiling view showing midline diastema between 11 and 21

In smile examination, the space between the maxillary central incisors is evident (Figure 2).

**Figure 3:**
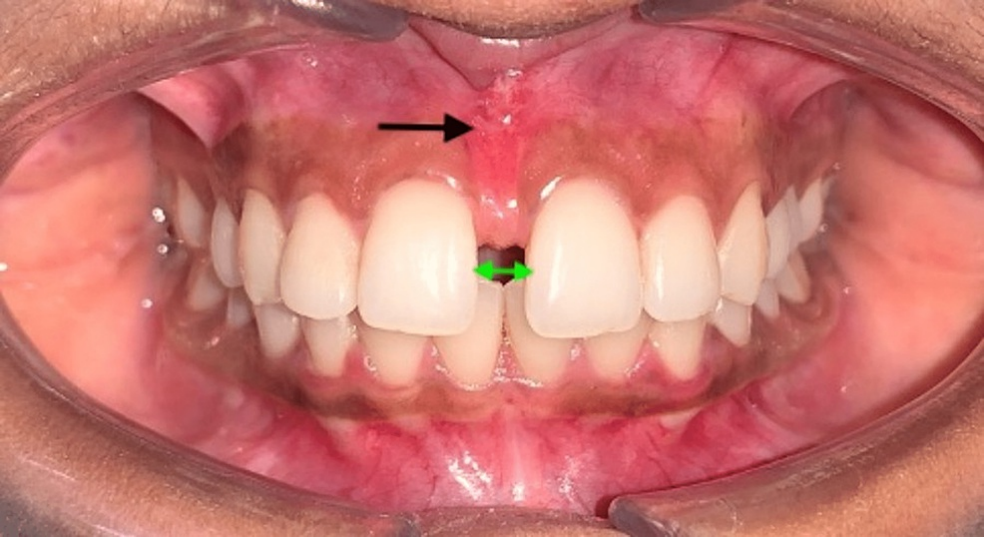
Pretreatment intraoral frontal view revealing a frenectomy of the labial frenum (black arrow) and a 4-mm midline diastema (green arrow)

An intraoral examination revealed that there was a midline gap of 4 mm between the 11 and 21 (Figure 3, green arrow). A Vernier caliper was utilized to measure the interdental distance that exists between the maxillary central incisors (Aerospace Digital Vernier Caliper, India). The frenectomy was conducted prior to the patient's visit to the orthodontics department, demonstrating that the high frenum attachment was the source of the midline diastema in this patient (Figure 3, black arrow).

**Figure 4:**
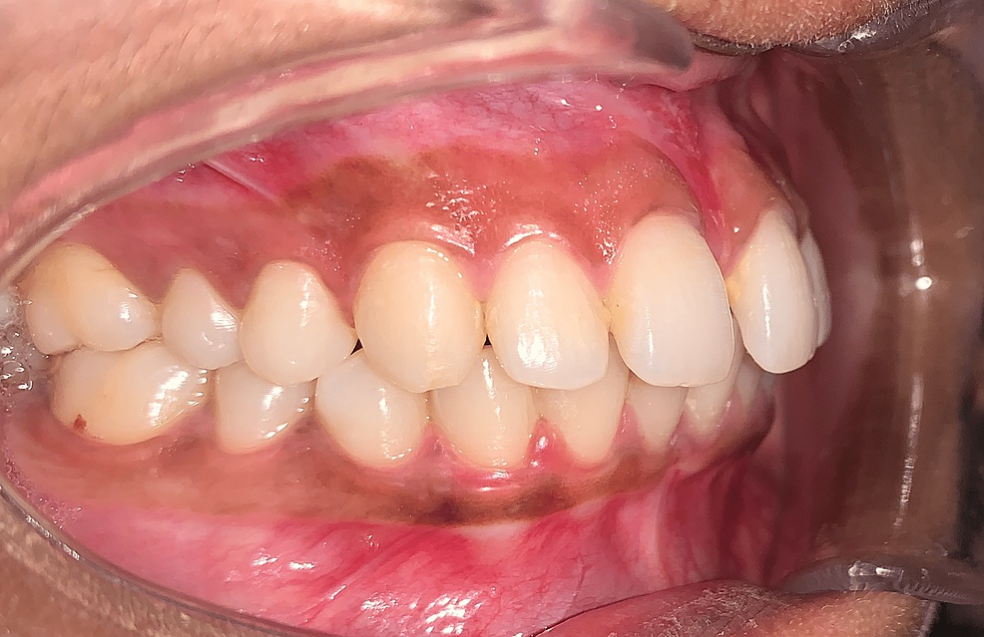
Pretreatment right lateral view showing Angle's class I molar and canine relation

**Figure 5:**
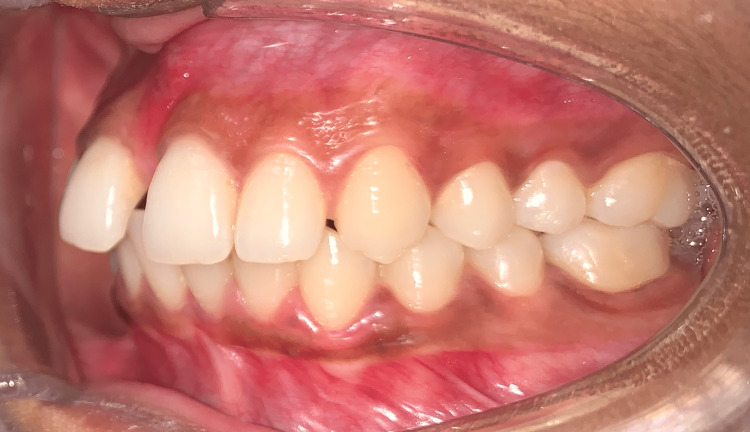
Pretreatment left lateral view showing Angle's class I molar and canine relation

There was a 4-mm overjet and an overbite present. The molar and canine were of Angle's class I relation on both sides (Figure 4 and Figure 5).

Treatment progress

The case was started with full-mouth oral prophylaxis to remove plaque and smears on the labial surface of the teeth. Phosphoric acid (37% concentration) was used to etch the teeth. First, only two maxillary central incisors were bonded with McLaughlin-Bennett-Trevisi (MBT) 0.018" slot brackets using Transbond composite resin (3M India Limited, Bangalore, India). The bracket was positioned using an MBT bracket positioning gauge. The bracket was placed using an MBT chart. A light cure was used to cure the brackets for 40 seconds. The "M" spring was chosen to close the midline void.

Construction of the "M" Spring

**Figure 6:**
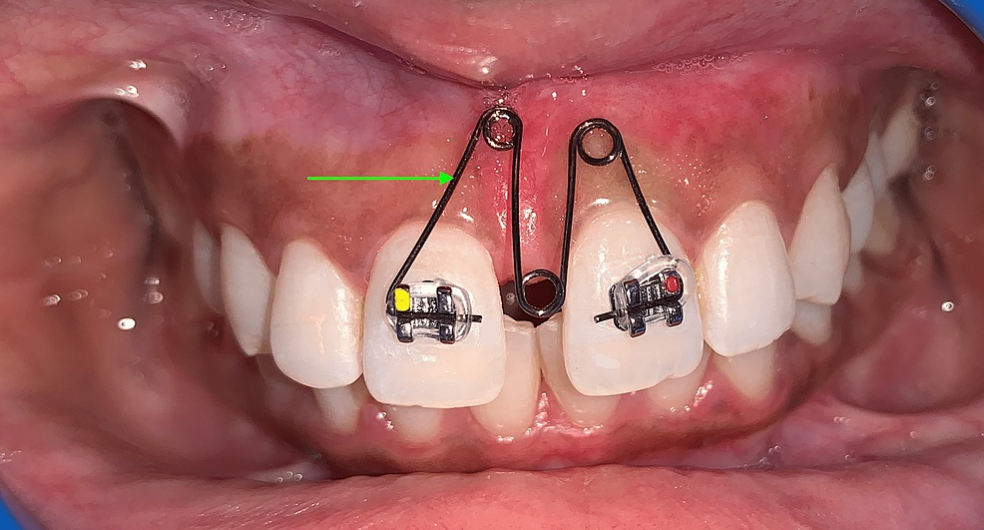
Maxillary central incisors loaded with M spring (green arrow)

The M spring is comprised of three circular loops, each measuring approximately 3-4 mm in diameter, with one located in the middle and the other two located adjacent (labial loop) to the middle loop. Labial loops were positioned 5-6 mm above the maxillary bracket. It was meticulously designed to stay out of the way of the labial sulcus and any other delicate soft tissues in the area. During activation, the active arm is given a 45-degree inward bend so that it completely rests in the bracket slot. This is necessary because the active arm needs to be inserted into the slot. The M coil spring was made out of 0.016" round AJ Wilcock stainless steel (SS) wire (AJ Wilcock, Victoria, Australia) (Figure 6, green arrow).

**Figure 7:**
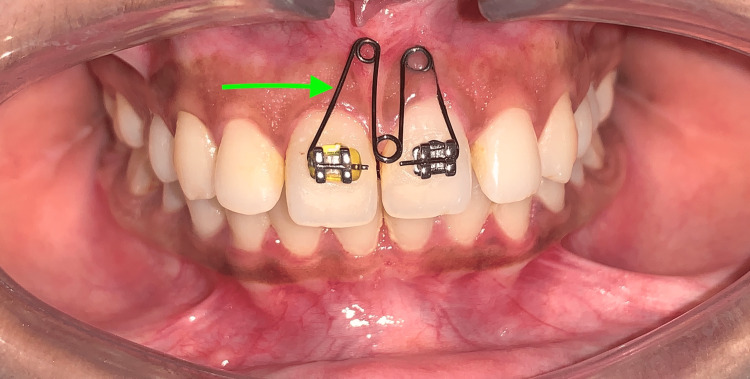
Midline space closer to the M spring (green arrow)

The midline diastema was successfully closed after four weeks of treatment with an "M" spring with brackets bonded only to the maxillary central incisors (Figure 7).

**Figure 8:**
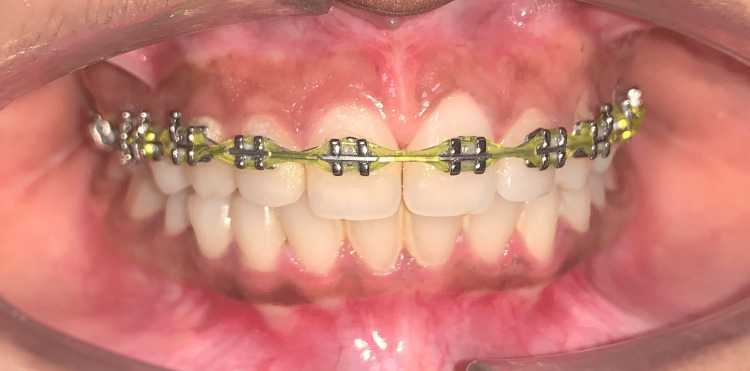
SS wire (17 × 25) in the upper arch with E-chain from canine to canine SS: stainless steel

There was a distance of 1 mm between the maxillary central and lateral incisors. The problem was caused by the tipping of both maxillary central incisors. In the remaining region, an MBT bracket was bonded to the upper arch, and a wire sequence consisting of 16 × 22 nickel-titanium (NITI), 16 × 22 stainless steel (SS), and 17 × 25 SS was placed for one month each (Figure 8).

**Figure 9:**
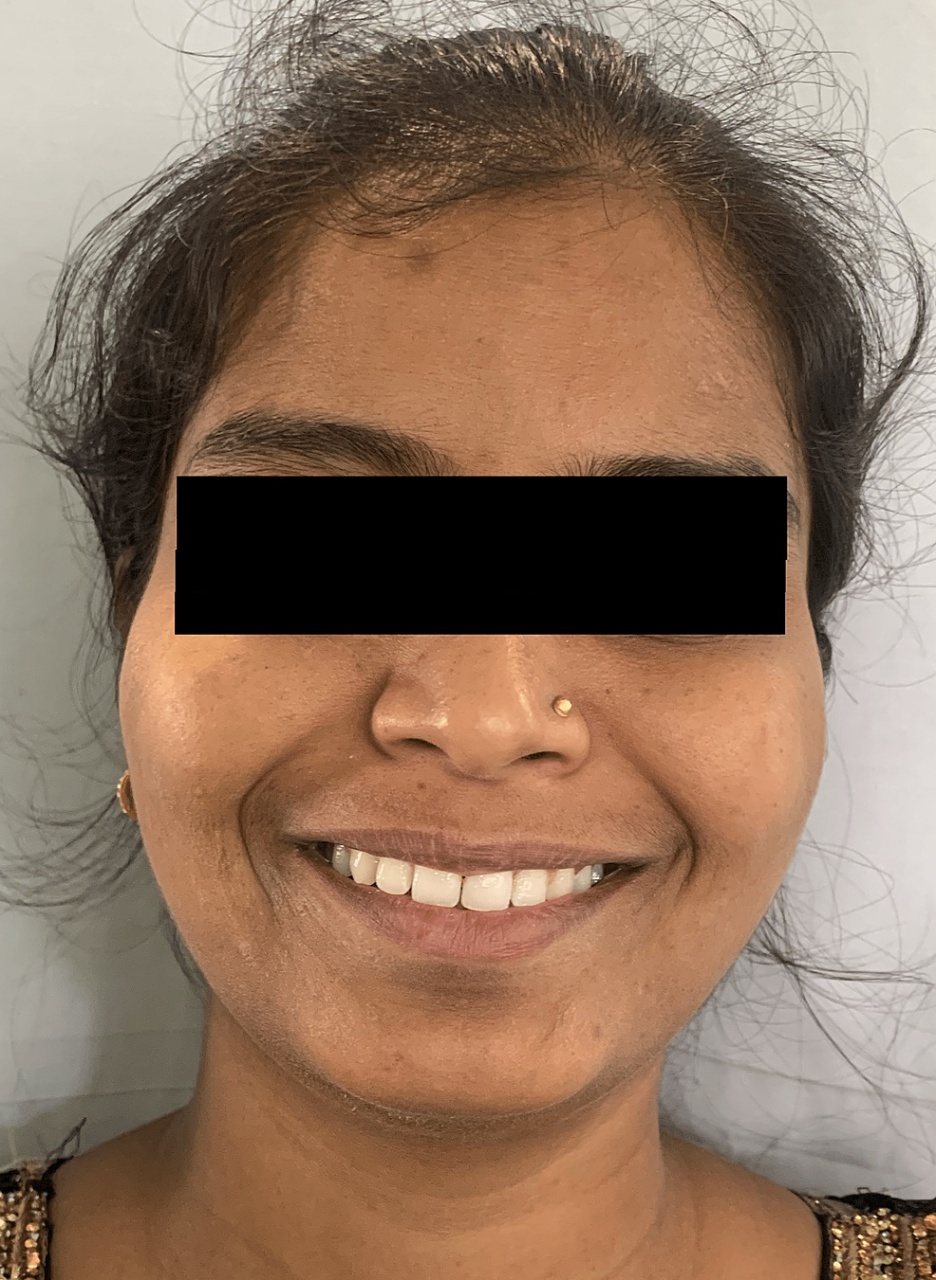
Posttreatment extraoral smiling view

**Figure 10:**
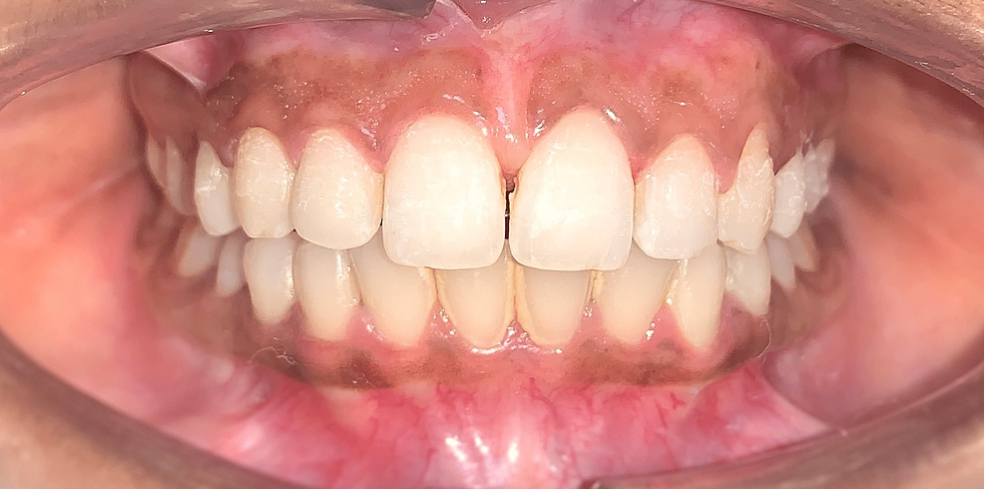
Posttreatment intraoral frontal view

**Figure 11:**
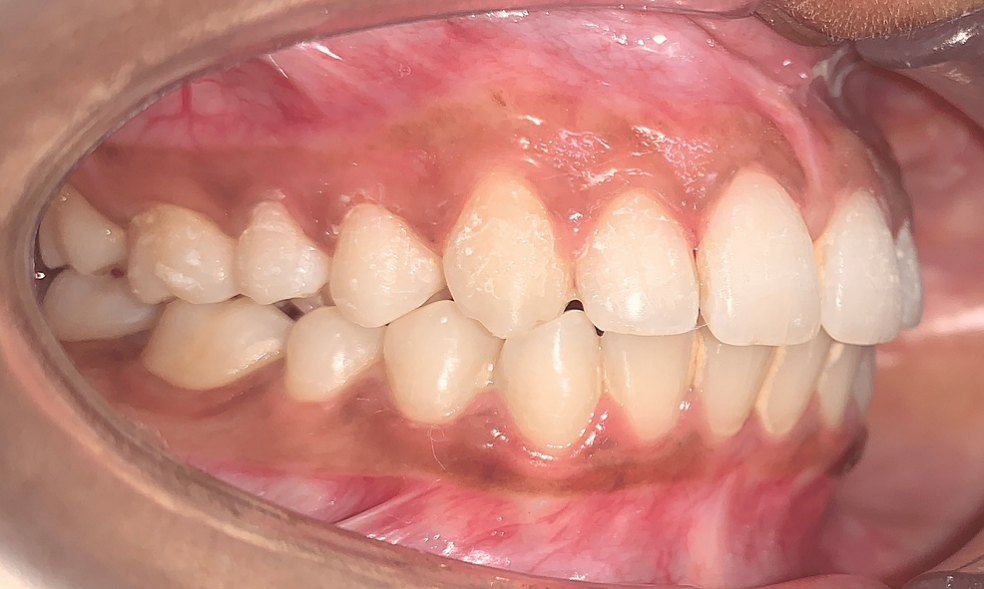
Posttreatment intraoral right lateral view

**Figure 12:**
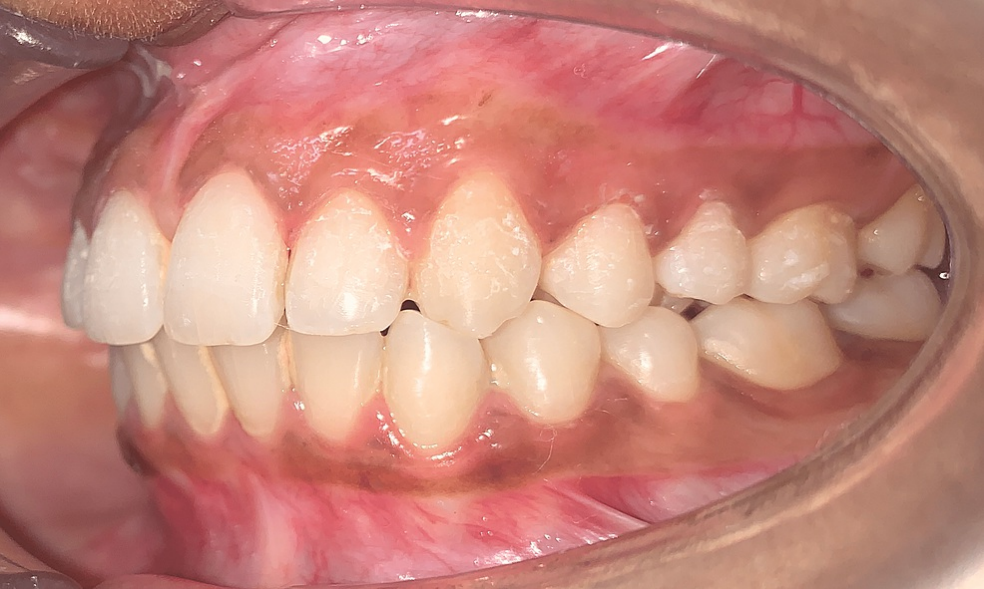
Posttreatment intraoral left lateral view

After a period of four months, all the remaining treatment was finished. In the maxillary arch, from canine to canine, a retainer that is fixed in place was placed (Figures 9-12).

## Discussion

Among young adults, esthetic consideration has increased with the passage of time. Patients who have midline diastemas have unesthetic smiles. Even the confidence of patients is reduced due to being bullied. A maxillary midline diastema is a common dental issue that manifests itself as an empty space between the incisors in the center of the upper jaw. There are a number of etiologic causes that have been identified for midline diastema. These include shifts in tooth material and jaw size, habits, congenitally absent lateral incisors, midline illnesses, peg laterals, and a number of other possibilities.

When treating patients with midline gaps, orthodontists frequently use a variety of metals. These metals are used to create a representation of the beautiful, natural smile that a human possesses. The characteristics and cross-sections of the metals that are used can vary. Prior to therapy, the wire needs to be chosen on the basis of its properties and measurements to achieve control over relapse. To fill in the gap in the midline, closed coil springs, E-chain, and various other types of wire are utilized.

Orthodontic closure of the midline diastema is categorized as follows: a treatment that involves a movement of the incisor by mesial tipping, a treatment method that involves the incisor's bodily movement, a treatment method that involves overjet reduced to its normal, and elimination of the space between the teeth as part of a more comprehensive orthodontic treatment plan.

In some cases, only the central incisors need to be moved to close the spaces. For patients who have good posterior occlusion, the diastema can be closed easily with sectional wire by forming orthodontic appliances. The sectional wire for space closer is either in the shape of a U or a V, and the closing loops are of the double-helix type incorporated in it. They are either connected directly bonded to the incisors or are bonded to the lingual surface or they are banded to the central incisors. In some cases, bodily movement of the incisors may be required to close a diastema. Fully bonded orthodontic brackets are used for leveling and alignment; then, the space closer steps are done. However, segmental archwire techniques are a viable option to consider if time or expense constraints make this type of treatment impractical or if the diastema is the only malocclusion that requires treatment. In this particular case (for bodily movement), brackets are bonded directly onto the four maxillary incisors, and a sectional wire of 0.018" stainless steel wire is placed. An elastic chain should be positioned such that it runs from the mesial wing of one lateral incisor bracket to the mesial wing of the other lateral bracket. This should be done by passing the chain or thread through the brackets of the centrals.

In the above case, the mechanics utilized tooth-tipping ideas proposed by Beggs in conjunction with lighter pressures applied via a circular archwire. Subsequently, root uprighting and torque management were accomplished through the utilization of pre-adjusted edgewise mechanics in conjunction with a rectangular archwire. When using the light archwire method, teeth can be moved simply by being tipped in the desired direction. When the round wire was used, there was one point of contact in the bracket slot that caused the central incisors to tip in a more mesial direction, which cause the midline diastema closure. Edgewise brackets with rectangular wires are pre-adjusted to manage the torque and root uprighting without causing any discomfort to tooth roots or adjacent tissues, as well as without causing any damage to the teeth. After treating a patient's midline diastema, orthodontists frequently face challenges with relapse. It is possible to avoid this problem by making use of retainers that are fixed in place rather than removable ones. The retention protocol should be adopted according to the size of the midline diastema to be closed as well as its underlying cause.

## Conclusions

During the mixed dentition stage of normal dental development, a midline diastema is typically present as part of the tooth development process known as "the ugly duckling stage." However, a diastema can be caused by a number of different factors, and it may require attention. Oral habits, muscular imbalances, physical impediments, abnormal maxillary arch structure, and various dental anomalies are some of the other etiologies associated with diastemas. Diastema treatment that is successful requires both an accurate diagnosis of the condition's underlying cause and treatment that is tailored to that particular root cause. A proper diagnosis should include taking a patient's medical and dental history, performing radiographic and clinical examinations, and perhaps even measuring the size of the patient's teeth. Modalities of treatment that are appropriate have been outlined. When trying to achieve satisfactory results, timing is frequently an important factor. After a proper diagnosis has been made and sufficient growth of the central incisors has occurred, it is typically possible to begin the process of removing the etiologic agent. However, if the space between the teeth is greater than 1.8 mm, even after the eruption of the lateral incisors, then orthodontic treatment will be required. Treating maxillary midline diastema with a sectional archwire by incorporating a loop in the shape of M has been shown to be an effective and innovative method. Both the amount of time that must be spent chairside and the number of supplies that are required are reduced as a result of this. In addition to this, the period of time required for the treatment has been decreased while maintaining the same high level of dependability in the outcomes. The retention protocol ought to vary according to the magnitude of the midline diastema as well as its underlying cause.
